# CNS-Wide over Expression of Fractalkine Improves Cognitive Functioning in a Tauopathy Model

**DOI:** 10.1007/s11481-018-9822-5

**Published:** 2018-11-29

**Authors:** Dylan J. Finneran, Dave Morgan, Marcia N. Gordon, Kevin R. Nash

**Affiliations:** 1 Department of Molecular Pharmacology & Physiology, Morsani College of Medicine, University of South Florida, Byrd Alzheimer’s Institute, 4001 E Fletcher Ave, Tampa, FL 33617 USA; 2 Translational Science and Molecular Medicine, Michigan State University, GRRC, 400 Monroe Ave. NW, Grand Rapids, MI 49503 USA

**Keywords:** Alzheimer’s disease, Tauopathy, Fractalkine, CX3CL1, Microglia

## Abstract

Accumulating evidence increasingly implicates regulation of neuroinflammation as a potential therapeutic target in Alzheimer’s disease and other neurodegenerative disorders**.** Fractalkine (FKN) is a unique chemokine that is expressed and secreted by neurons and reduces expression of pro-inflammatory genes. To further demonstrate the utility of agents that increase FKN signaling throughout the central nervous system as possible therapies for AD, we assessed the impact of soluble FKN (sFKN) over expression on cognition in tau depositing rTg450 mice after the onset of cognitive deficits. Using adeno-associated virus serotype 4, we infected cells lining the ventricular system with soluble FKN to increase FKN signaling over a larger fraction of the brain than achieved with intraparenchymal injections. We found that soluble FKN over expression by cells lining the ventricles significantly improved cognitive performance on the novel mouse recognition and radial arm water maze tasks. These benefits were achieved without detectable reductions in tau hyperphosphorylation, hippocampal atrophy, or microglial CD45 expression. Utilizing qPCR, we report a significant increase in *Vegfa* expression, indicating an increase in trophic support and possible neovascularization in AAV-sFKN-injected mice. To our knowledge, this is the first demonstration that FKN over expression can rescue cognitive function in a tau depositing mouse line.

**Graphical Abstract:**
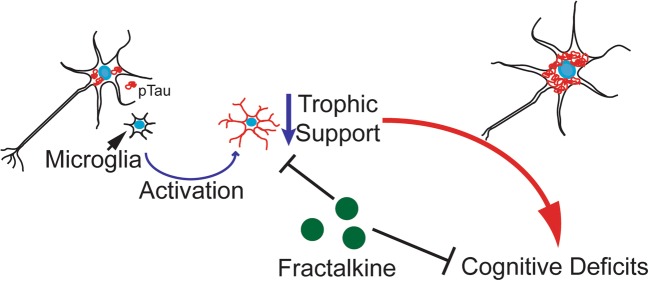
Regulating neuroinflammation is an attractive therapeutic target for Alzheimer’s disease. Microglial activation can not only drive pathology but also accelerate cognitive decline. The chemokine fractalkine regulates the microglial phenotype, increasing trophic support of neurons, and significantly improving cognitive functioning in the rTg4510 mouse model of tauopathy.

Regulating neuroinflammation is an attractive therapeutic target for Alzheimer’s disease. Microglial activation can not only drive pathology but also accelerate cognitive decline. The chemokine fractalkine regulates the microglial phenotype, increasing trophic support of neurons, and significantly improving cognitive functioning in the rTg4510 mouse model of tauopathy.

## Introduction

Fractalkine (CX3CL1; FKN) is unique among chemokines because it is the only member of the CX3C motif family and has a one-to-one relationship with its receptor, CX3CR1 (Bazan et al. 1997; Imai et al. 1997; Pan et al. 1997). Produced as a transmembrane protein, FKN can undergo proteolytic cleavage by a disintegrin and metalloprotease (ADAM) 10/17 or cathepsin S to produce a secreted, soluble form of the protein (sFKN) (Garton et al. 2001; Hundhausen et al. 2003; Jones et al. 2013). There is evidence that the membrane-associated (full-length) and ectodomain (soluble) forms of FKN may have different functional roles and activate the receptor differently (Clark and Malcangio 2012; Kim et al. 2011). In the periphery, the full-length form is important for monocyte adhesion to endothelial cells while the soluble form may act as a chemoattractant for lymphocytes and monocytes (Imai et al. 1997). In the central nervous system (CNS), FKN is produced by neurons and its receptor is expressed only on microglia (Cardona et al. 2006; Harrison et al. 1998). FKN signaling in the CNS blunts microglial activation, reducing production of pro-inflammatory cytokines such as interleukin (IL)-1β, IL-6 and tumor necrosis factor-α (Lyons et al. 2009).

Fractalkine’s role in neurodegenerative diseases has been of considerable interest in the last few years. Initial work disrupting FKN signaling in animal models (receptor or ligand knockouts) demonstrated increased Parkinson’s disease pathology, tauopathy, and amyotrophic lateral sclerosis (Bhaskar et al. 2010; Cardona et al. 2006), suggesting that dysregulation of microglial activation can result in worsening of disease pathology and neurodegeneration. However, disrupting FKN signaling in amyloid-depositing mice showed amelioration of the pathology due to increased microglial phagocytosis of the extracellular amyloid plaques (Lee et al. 2010b). Conversely, studies investigating the over expression of FKN showed opposite results to disruptions in signaling, generally leading to favorable improvements in disease pathology. The soluble form of FKN was shown to be beneficial in multiple models of Parkinson’s disease (Morganti et al. 2012; Nash et al. 2015; Pabon et al. 2011). In Alzheimer’s disease, we reported that adeno-associated virus (AAV) over expression of sFKN in the hippocampus of the rTg4510 mouse model of tauopathy resulted in reduced tau pathology, amelioration of neuron loss, and reduction of microgliosis. However, there was no improvement in cognitive performance after sFKN over expression, which may be attributed to the limited distribution of the treatment which was targeted to the dentate gyrus of the hippocampus (Nash et al. 2013).

Here, we further investigate the therapeutic ability of sFKN by addressing two questions: does a more global distribution of CNS sFKN expression have a greater cognitive impact and can increased sFKN expression have an impact in animals that already have advanced tau pathology? The latter would be more clinically informative because most AD patients would have significant tau pathology at the time of their diagnosis. To achieve this, we used a novel delivery method into five-month old rTg4510 animals, which present with insoluble tau and cognitive deficits (Dickey et al. 2009; Santacruz et al. 2005). We utilized the unique capability of AAV serotype 4 to infect cells lining the ventricular system, combined with the soluble nature of our therapeutic gene product, to distribute sFKN to the CNS via secretion into the CSF. This has been achieved with other secreted proteins (Davidson et al. 2000b; Liu et al. 2005; Tenenbaum et al. 2004). We demonstrate here that elevated sFKN expression via AAV4, during late stage disease pathology, can partially rescue behavioral deficits but does not appear to alter disease progression.

## Methods

### Adeno-Associated Virus Production

The ectodomain (amino acids 1–336 containing the chemokine domain and mucin-like stalk) of mouse fractalkine was isolated from mouse cDNA and cloned into pTR2-MCS vector at the *Age* I and *Nhe* I sites as described previously (Morganti et al. 2012; Nash et al. 2013)**.** This vector contained the AAV2 terminal repeats and hybrid cytomegalovirus-chicken β-actin (CBA) promoter. A C-terminal hemagglutinin (HA)-tag was added for protein detection. rAAV4 particles were generated as described previously (Carty et al. 2010) and quantified using a dot-blot method with a non-radioactive biotinylated probe for fractalkine generated by polymerase chain reaction (Burger and Nash 2016; Nash et al. 2013).

### Transgenic Mice and Breeding

Animal experiments were conducted in accordance with the National Institute of Health Guide and Use of Laboratory Animals and were approved by the Institutional Animal Care and Use committee of the University of South Florida. Parental mutant tau and tetracycline-controlled transactivator protein strains were maintained separately and bred to produce rTg4510s and littermate nontransgenic (NonTg) and tetracycline-controlled transactivator protein (tTA) mice as previously described (Santacruz et al. 2005). Littermate tTA and NonTg mice were used as behavioral controls, as we observed modest differences in performance between tTA and NonTg mice. Study animals were given food and water ad libitum and maintained on a 12-h light/dark cycle.

### Surgical Procedure and Tissue Collection

Immediately before surgery, mice were weighed and anesthetized with isoflurane. Surgeries were performed using a stereotaxic apparatus using convection enhanced delivery as described previously (Burger and Nash 2016; Carty et al. 2010; Nash and Gordon 2016). Animals receiving AAV4 sFKN (3.4 × 10^12^ vg/mL) were injected bilaterally into the lateral ventricles with 5 μL of virus in sterile PBS per site (coordinates from bregma: −0.4 mm anteroposterior, ±1.0 mm lateral, & -2.4 mm vertical). Control animals received AAV9 UF11 (5 × 10^12^ vg/mL) expressing green fluorescent protein (GFP) were injected as described previously (Carty et al. 2010).

Three months post-surgery, mice were weighed and overdosed with pentobarbital (200 mg/kg). CSF was collected as described (Liu and Duff 2008) and mice were perfused with 25 mL of 0.9% normal saline. Brains were collected immediately after perfusion. The right hemisphere was dissected and frozen on dry ice for biochemical analysis. The left hemisphere was immersion fixed in 4% paraformaldehyde for 24 h. The fixed hemisphere was cryoprotected with successive incubations in 10%, 20%, and 30% sucrose solutions for 24 h in each solution. Brains were frozen on a cold stage and sectioned horizontally (25 μm thick) on a sliding microtome and sections were stored in PBS with 10 mM sodium azide at 4 °C. For rTg4510 mice, every twelfth section was 50 μm.

### Tissue Homogenization, ELISA, and Western Blotting

Anterior cortex and hippocampal samples were homogenized in TBS with protease inhibitor cocktail (Sigma Aldrich, St. Louis, MO, USA; Cat. No. P8340), phosphatase inhibitor cocktails II and III (Sigma Aldrich, St. Louis, MO, USA; Cat. Nos. P5726 & P0044, respectively), and Benzonase (Sigma Aldrich, St. Louis, MO, USA; 25 U/mL final concentration) at 10 vol/wt of tissue. Tissue was homogenized with a rotating pestle and briefly sonicated (3 × 3 s). The samples were centrifuged for 10 min at 10,000 x *g* at 4 °C. An aliquot (15 μL) of the resulting supernatant was taken for FKN ELISA. The pellet was resuspended in the remaining supernatant and detergents added to a final concentration of 0.01% SDS, 0.1% NP40, and 0.05% sodium deoxycholate. Samples were then centrifuged at 40,000 x *g* for 30 min at 4 °C. The resulting pellet was resuspended in 70% formic acid (2 μL/mg tissue) and incubated for 60 min at room temperature. An equal volume of 1 M Tris pH 7.5 was added and the sample was neutralized to pH 7.5 with NaOH if needed. The soluble fraction was taken for Western blotting and total protein concentration was determined by Pierce BCA protein assay (ThermoFisher Scientific, Walthman, MA, USA). For Western blotting, 1 μg of protein was loaded for each sample. H150 (total tau; Sigma), anti-phospho-Ser199/202 tau (pSer199/202, Anaspec, Fremont, CA, USA), and anti-phosphoSer396 tau (pSer396, Anaspec, Fremont, CA, USA) were used to assess tau pathology. All signals were normalized to β-actin (ThermoFisher Scientific, Walthman, MA, USA). For the insoluble fraction, which does not contain actin, an equal volume of each sample was loaded. A fractalkine ELISA was obtained from R&D Systems and the manufacturer’s protocol was followed.

### Behavioral Assessment

All behavioral tasks were conducted by an observer blinded to treatment condition and genotype of the mice. The open field was used as a general measure of activity and anxiety. Animals were recorded for 15 min in a 40 cm by 40 cm open field box with video tracking software (ANY-Maze, Stoelting, Wood Dale, IL, USA). General activity levels were evaluated by distance traveled in the open field.

Each animal was placed for a single, 5-min trial in a Y-maze and activity was recorded (ANY-Maze, Stoelting, Wood Dale, IL, USA). The number of arm entries and spontaneous alternation, entering each arm in sequence without repetition, was expressed as a percentage as previously described (Brownlow et al. 2014).

Short term memory was evaluated by the novel object recognition task. Two objects, similar in scale to the mice, were placed in the 40 cm × 40 cm open field arena approximately 3–5 cm from the outer wall. Each animal was given three 5-min familiarization trials with a 5-min inter-trial interval. On the fourth trial, one of the objects was switched for a novel object. Animals were given five minutes to explore the objects and their activity was recorded (ANY-Maze, Stoelting, Wood Dale, IL, USA). The objects and arena were cleaned between trials with 10% ethanol to minimize olfactory cues. Working memory was evaluated by measuring the time spent with familiar object and novel object on the final trial.

To overcome the disinterest the mice showed in the objects, the mice were also tested in a variant of the NOR task using unfamiliar mice as the stimuli as previously described (Brownlow et al. 2014). Briefly, the test mouse was given one 5-min habituation trial to its environment. It was then introduced to two sex-matched bait mice, located in the left or right chamber of the three-chambered arena. The test mouse had two five-minute trials with a five-minute inter-trial interval to familiarize itself with the bait mouse. On the fourth trial, one of the bait mice was switched for a novel bait mouse. The test mouse was given another five minutes to explore the arena and its activity was recorded (ANY-Maze, Stoelting, Wood Dale, IL, USA). Preference for the novel mouse was assessed by measuring time spent in the chamber of the arena containing the novel mouse.

Radial arm water maze (RAWM) has been described in detail, including sample score sheets, previously (Alamed et al. 2006). The radial arm water maze contained six arms radiating from an open central area with a hidden escape platform located at the end of one of the arms. Around the pool, several extra-maze cues were hung to allow for spatial navigation. On each trial, the mouse was allowed 60 s to find the platform. The platform was located in the same goal arm on each trial. On day one, the mice were given 15 trials alternating between a visible and hidden platform. On day two, mice were given 15 additional trials, all with a hidden platform. The start arm was varied for each trial, forcing mice to rely on the extra-maze spatial cues to find the platform instead of procedural memory. The goal arm for each mouse was different to avoid odor cues revealing the platform location. Entry into an incorrect arm (all four limbs in the arm) was counted as an error. Failure to enter an arm for 15 s was also counted as an error. The errors of blocks of three trials were averaged for data analysis. Mice that made one or fewer errors on the last block of Day 2 were considered to have learned the platform location.

On the third day, a reversal trial was performed with the goal arm moved 180° across the pool. The mice were again given 15 trials, all with the hidden platform, to learn its new location. On the fourth day, the arm insert was removed from the pool, the extra-maze cues were taken down, and the platform was raised above the surface of the water with a flag attached to confirm that all mice were able to see and capable of ascending the platform. Latency to find and ascend the platform was recorded with a maximum swim time of 60s.

To assess thermal hyperalgesia, mice were placed on a hot plate at 50 °C. Latency to hind paw withdrawal was measured (Woolfe and Macdonald 1944).

### Immunohistochemistry

Six to eight sections approximately 200 μm apart spanning the hippocampus were chosen for analysis. Immunohistochemical experiments were performed as described previously (Gordon et al. 2002). Briefly, sections for each animal were placed into a multisample staining tray. Endogenous peroxidases were blocked (10% methanol, 3% hydrogen peroxide in PBS for 15 min) and tissue was permeabilized (0.2% lysine, 0.1% Triton X-100 in PBS for 30 min). Sections were incubated overnight in appropriate primary antibody: anti-pSer396 (Anaspec, Fremont, CA, USA); anti-CD45 (ThermoFisher Scientific, Walthman, MA, USA); anti-IBA-1 (Wako, Richmond, VA, USA); or HRP-conjugated anti-HA (Roche, Indianapolis, IN, USA). Sections were washed three times in PBS, then incubated for two hours with corresponding biotinylated secondary antibody (Vector Laboratories, Burlingame, CA, USA), if necessary. The tissue was again washed and incubated with Vectastain Elite ABC Kit (Vector Laboratories) for enzyme conjugation. Finally, sections were stained using 0.05% diaminobenzidine and 0.03% hydrogen peroxide for five minutes. Each immunohistochemical assay omitted some sections from primary antibody incubation to evaluate nonspecific binding of the secondary. Sections were mounted onto slides, dehydrated, and coverslipped.

Gallyas staining was performed as previously described (Lee et al. 2010a). Staining was performed on pre-mounted tissue sections that had been dried for a minimum of 24 h. Prior to staining sections were rehydrated for 30 s. Slides were treated with 5% periodic acid for five minutes, washed with water, and incubated sequentially in silver iodide (1 min) and 0.5% acetic acid (10 min) prior to being placed in developer solution (2.5% sodium carbonate, 0.1% ammonium nitrate, 0.1% silver nitrate, 1% tungstosilicic acid, 0.7% formaldehyde). Slides were incubated in developer solution until color developed after which they were placed in 0.5% acetic acid to stop the reaction (3 min). Slides were incubated in 0.1% gold chloride solution (5 min), washed with water, and incubated in 1% sodium thiosulphate solution (5 min). After a final wash, slides were dehydrated and coverslipped.

Stained sections were imaged using a Zeiss Axioscan.Z1 scanning microscope and Neurocyte IAE software (created by Andrew Lesniak) was used for analysis. The area of positive staining in the hippocampus was analyzed. The software used hue, saturation, and intensity to segment the images and these values were held constant for analysis of every section of every animal in each stain. These values were established on sections of high and low levels of staining to identify positive staining over background (Gordon et al. 2002).

### RNA Isolation and Real-Time PCR

RNA was isolated from posterior cortex using the Zymo Quick-RNA Miniprep Plus kit (Zymo Research, Irvine, CA, USA; cat. no. R1057) per the manufacturer’s recommended protocol. EXPRESS One-Step Superscript qRT-PCR Kit (cat. no. 11718200) and TaqMan Gene Expression Assays were purchased from ThermoFisher (Walthman, MA, USA). Primers for the target genes Tnf (Mm00443256_m1), Il1b (Mm00434228_m1), Il6 (Mm00446190_m1), Il12a (Mm00434169_m1), Il10 (Mm01288386_m1), Vegfa (Mm00437306_m1), Il4 (Mm00445259_m1), Marco (Mm00440265_m1), Itgam (Mm00434455_m1), CD68 (Mm03047343_m1), Arg1 (Mm00475988_m1), Nos2 (Mm00440502_m1), C1qa (Mm00432142_m1), Itgax (Mm00498701_m1), and the housekeeping gene Pgk1 (Mm00435617_m1) were used per the manufacturer’s recommended protocol. Fifty nanograms of RNA were loaded per well and data collected in a Bio-Rad CFX96 thermal cycler (50 °C for 15 min, 95 °C for 2 min, 40 cycles of: 95 °C for 15 s, 60 °C for 1 min). Data was analyzed using the ΔΔC_T_ method (Schmittgen and Livak 2008).

### Statistical Analysis

Statistical analysis was performed using SPSS Statistics (IBM, Armonk, NY, USA). For behavioral tasks, all groups were assessed using a one-way analysis of variance (ANOVA) was performed with Fisher’s LSD post-hoc analysis. For the novel object recognition task, a two-tailed Student’s t test was used to compare time spent with the familiar object to time spent with the novel object on trial four. The novel mouse recognition task was analyzed with a two-way ANOVA and Fisher’s LSD with Group and Chamber as fixed factors. For tau western blotting and immunostaining, where there was no signal from mice lacking P301L tau expression, a two-tailed Student’s t test was utilized to compare sFKN-treated mice to GFP-injected controls. Immunostaining for CD45 and IBA-1 was analyzed with a one-way ANOVA and Fisher’s LSD post-hoc analysis comparing all groups. The threshold for significance was set at *p* = 0.05.

## Results

### Intraventricular Injection of AAV4 Increased Soluble FKN

**Fig. 1:**
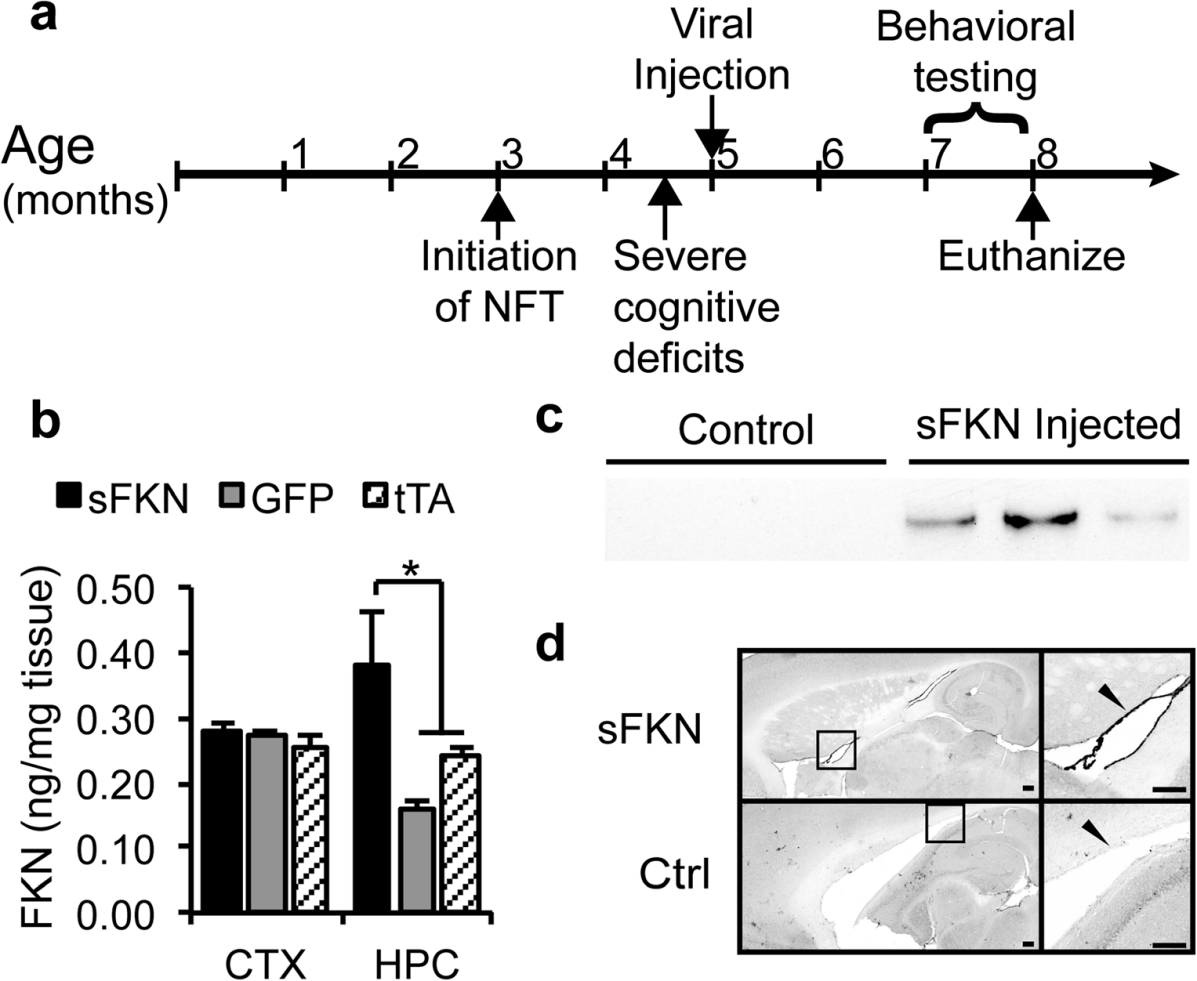
AAV4 over expressing sFKN transduced cells of the lateral ventricle leading to increased sFKN in the hippocampus**. a** Study design. Five-month old rTg4510s were injected with AAV4-sFKN (3.4 × 10^12^ vg/mL) and survived for two months prior to behavioral assessment. Tissue was collected at eight months of age. **b** A FKN ELISA showed increased sFKN in the hippocampus of animals injected with AAV4-sFKN compared to GFP-injected rTg4510 and uninjected tTA control mice (*n* = 9–12). **c** Anti-HA Western blot showed sFKN secretion into the CSF of AAV4-sFKN injected mice. **d** Representative images of sections stained for anti-HA showed positive staining in the ventricles of animals that received AAV4-sFKN, but not in control mice (scale bar = 250 μm). Data represented as mean ± SEM. * denotes *p* < 0.05 by Fisher’s LSD

Adeno-associated virus serotype 4 has been reported to readily infect cells lining the ventricular system in the CNS (Davidson et al. 2000b; Dodge et al. 2010; Liu et al. 2005; Tenenbaum et al. 2004). Here, we sought to take advantage of this AAV4 tropism to infect cells lining the ventricular system in order to increase the distribution of soluble fractalkine (sFKN) agonism throughout the CNS with minimal intracranial injections. Fig. 1a describes the timing of the FKN administration relative to assessment of behavioral, histological and biochemical endpoints. To confirm increased sFKN levels in the parenchyma, we performed an ELISA on soluble fractions of brain homogenate three months post-injection. Injection with AAV4 sFKN increased sFKN concentrations in the hippocampus but not the anterior cortex (Fig. 1b) compared with to control-injected rTg4510s. Staining for anti-HA shows positive staining in the lining of the ventricles in injected animals not present in uninjected control mice (Fig. 1d). We also observed recombinant sFKN expression in the CSF by Western analysis for HA present on the sFKN construct (Fig. 1c). Thus, AAV4 over-expressing sFKN delivered into the ventricles causes secretion of sFKN into the CSF, which diffuses into the hippocampus leading to supraphysiological levels of FKN.

### Increased Soluble FKN Expression Ameliorated Hyperactivity and Improved Cognition

**Fig. 2:**
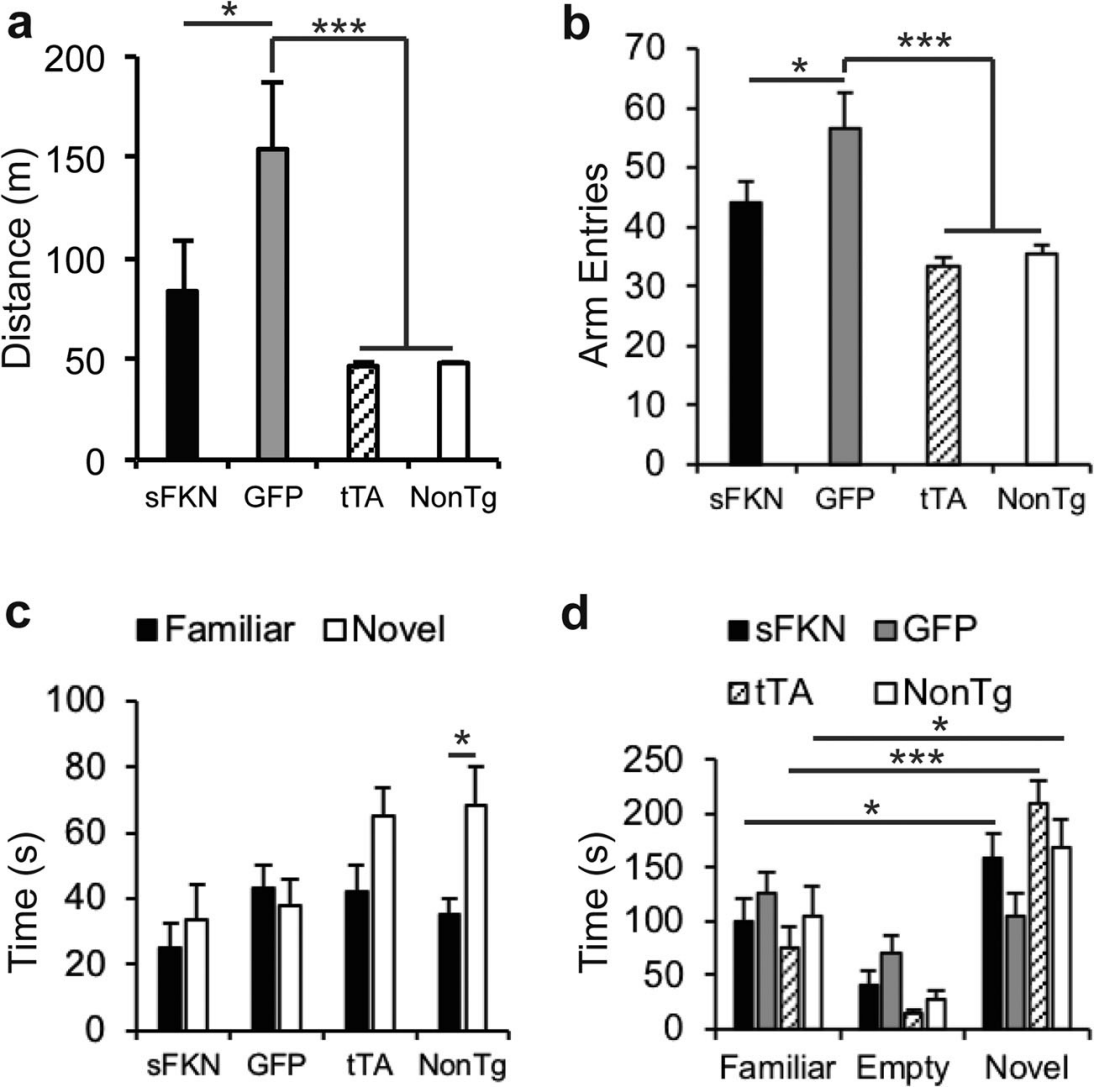
AAV4 sFKN over expression reduced hyperactivity and improved novel mouse recognition. **a** Distance travelled in open field was reduced in sFKN animals; **b** arm entries Y-maze were reduced in sFKN animals. Locomotor activity of tTA (hatched bars) and NonTg (open bars) mice was lower than locomotor activity in AAV-GFP-injected transgenic mice in both tests. **c** Nontransgenic mice showed a preference (increased interaction time) for the novel object over the familiar object in the novel object recognition test, but the other test groups did not show a preference. **d** Nontransgenic, tTA and AAV-sFKN-treated mice showed a significant preference for interacting with a novel mouse, but AAV-GFP-treated mice did not. Data represented as mean ± SEM, *n* = 9–12. * denotes *p* < 0.05; *** denotes *p* < 0.001 by Fisher’s LSD

rTg4510 mice have been shown previously to be hyperactive (Brownlow et al. 2013, 2014; Joly-Amado et al. 2016). We also observed increased locomotion in transgenic mice treated with control AAV injections compared with either tTA or nontransgenic uninjected mice (Fig. 2a, b). Increased soluble fractalkine signaling ameliorated this behavior with sFKN-treated mice both traveling a shorter distance in the open field (Fig. 2a) and entering fewer arms in the Y-maze (Fig. 2b) than GFP-injected control mice. We observed no significant differences between groups in spontaneous alternation in the Y-maze (data not shown). While sFKN over expression did not improve object recognition (Fig. 1c), it did significantly improve novel mouse recognition. Mice treated with sFKN spent more time interacting with the novel mouse than the familiar mouse while GFP-injected transgenic mice showed no preference for either the novel, familiar, or empty center chamber of the apparatus (Fig. 2d).

**Fig. 3:**
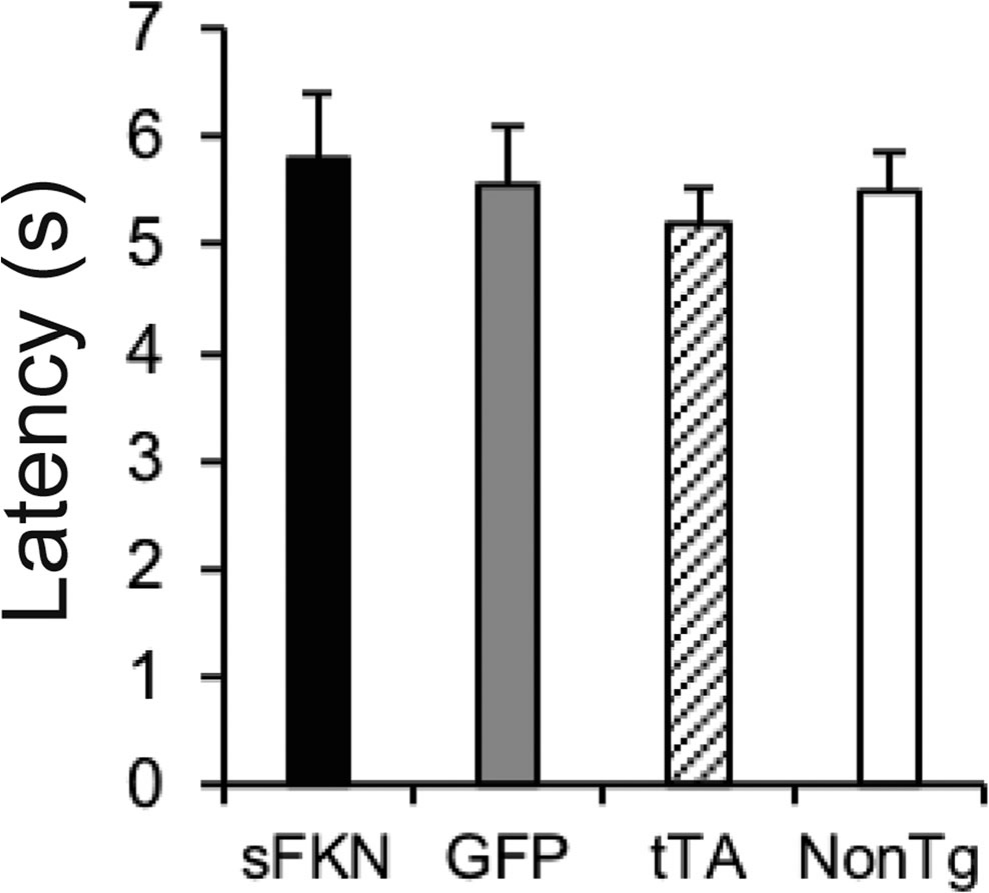
Over expression of the ectodomain of FKN did not cause thermal hyperalgesia. Graph of latency to withdraw hind paw from hot plate. Over expression of the ectodomain of FKN (with mucin-like stalk), did not affect latency to respond to heat after. Data represented as mean ± SEM, *n* = 9–12

Previous reports have implicated a truncated soluble variant of FKN generated by Cathepsin S cleavage in neuropathic pain (Clark and Malcangio 2012; Clark et al. 2007, 2009). To investigate if the entire ADAM 10/17 cleaved ectodomain of FKN may contribute to this, we assessed thermal hyperalgesia on a hot plate. Animals over expressing the entire ectodomain of FKN did not have a change in the latency to hind paw withdrawal (Fig. 3), indicating that they did not have a change in pain threshold. Nor did they exhibit any signs of adverse sickness behavior, such as hunching. Thus, we conclude the mice were not made hyperalgesic by the sFKN expression.

**Fig. 4:**
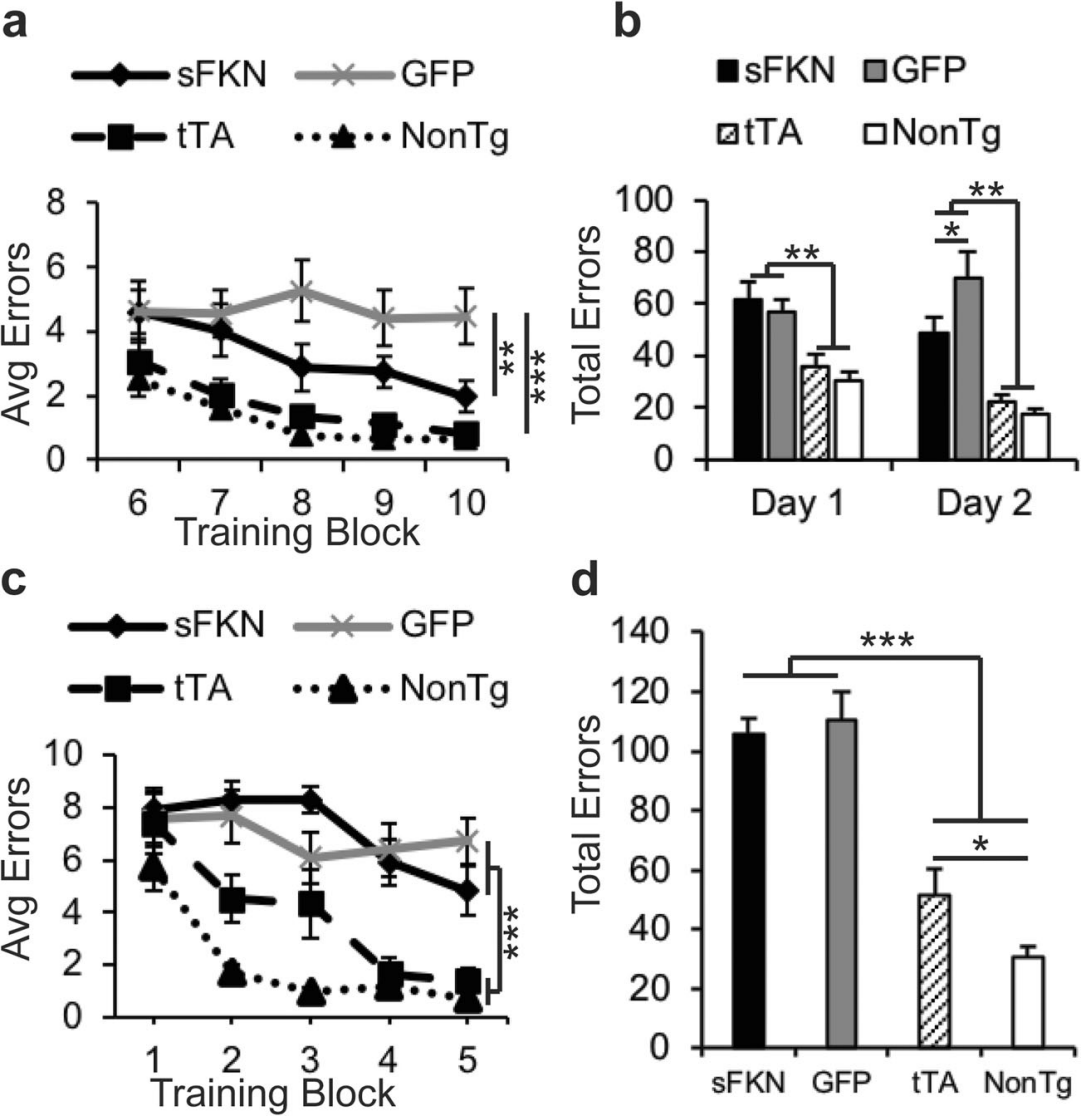
sFKN over expression improved performance on RAWM but not on Reversal learning. **a** Graph of errors made during day 2 training in RAWM. Over expression of sFKN reduced average errors made on the last block of day 2; **b** Graph of total errors made on day 1 and day 2 of RAWM. sFKN reduced the total number of errors made on Day 2 compared to AAV-GFP injected rTg4510s. **c** Errors made during reversal testing. **d** Graph of total errors made during reversal testing. sFKN over expression did not reduce average errors per block compared GFP animals. Data represented as mean ± SEM, *n* = 9–12. * denotes *p* < 0.05; ** denotes *p* < 0.01; *** denotes *p* < 0.001 by Fisher’s LSD

We have previously reported rTg4510 mice display a deficit in spatial learning and memory by five months of age. Using the radial arm water maze task, we observed that AAV-GFP-treated control transgenic mice displayed a larger number of errors per block compared with either tTA or nontransgenic mice (Fig. 4a), commit significantly more total errors on both day 1 and day 2 (Fig. 4b), and never attain criterion performance of <1 error per block by the end of day 2. In contrast, treatment with sFKN revealed a partial rescue in performance, with a smaller number of average errors on Block 10 and a significant reduction in errors made on Day 2 of testing compared to GFP-injected controls (Fig. 4a, b). However, sFKN over expression was not able to improve performance in the reversal portion of the task (Fig. 4c, d).

### Increased Soluble FKN Signaling Did Not Reduce Tauopathy

**Fig. 5:**
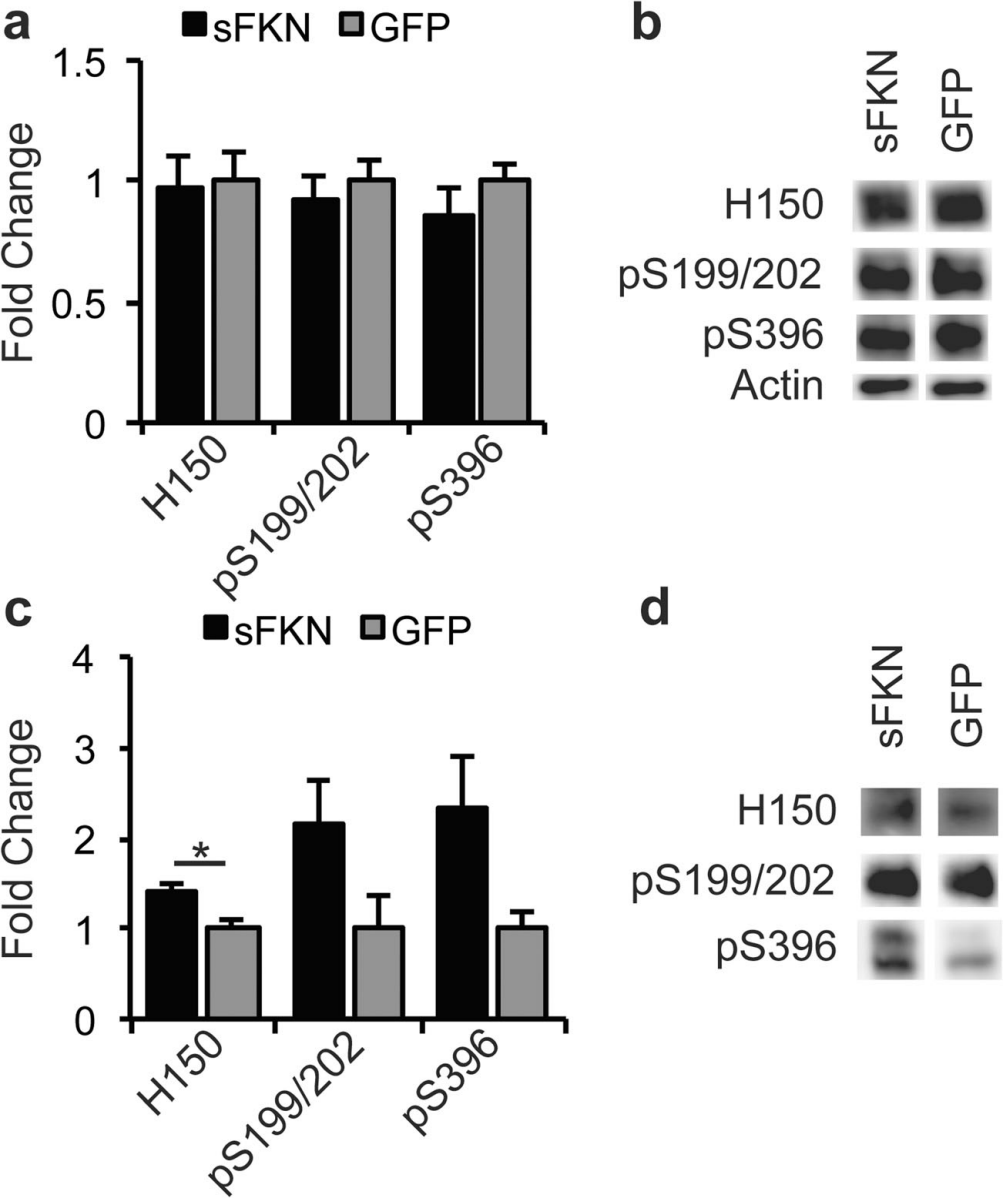
sFKN did not ameliorate tauopathy and increased insoluble total tau. **a** Graph of tau and phospho-tau levels from Western blot analysis. Soluble total tau (H150) and phospho-tau (pS199/202 & pS396) were unchanged by sFKN over expression. **b** Displays representative Western blots of monomeric tau at 55 kDa. **c** Graph of insoluble tau and phospho-tau levels from Western blot analysis. sFKN over expression increased insoluble total tau with trends for increased insoluble phospho-tau. **d** Displays representative Western blot images of monomeric tau at 55 kDa. Intensity of blots from all animals were averaged and converted to fold change for display. Data represented as mean ± SEM, *n* = 9–12. * denotes *p* < 0.05 by two-tailed Student’s t test

**Fig. 6:**
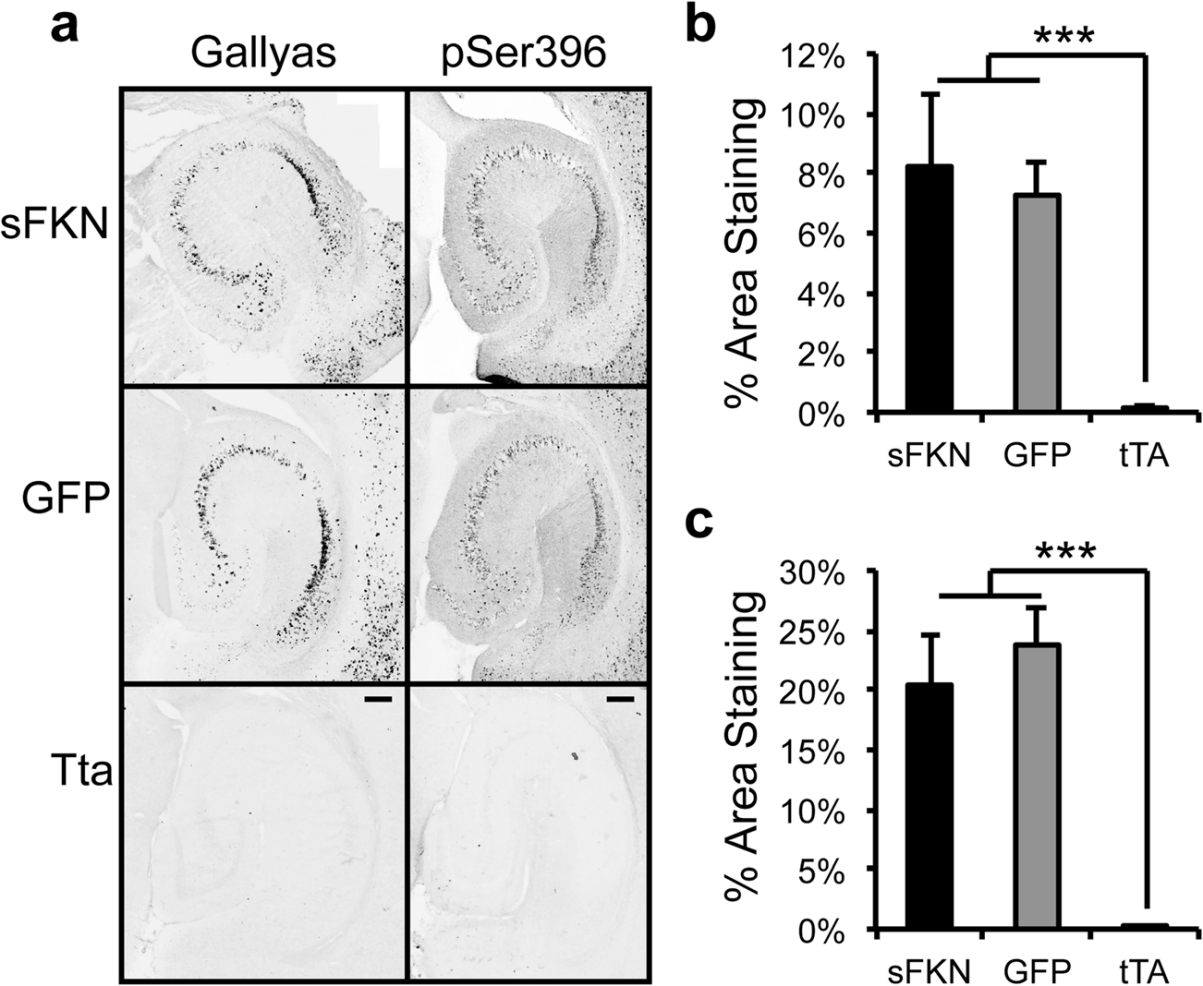
sFKN over expression did not reduce pSer396 phospho-tau or Gallyas-positive tau tangles. **a** Representative images of hippocampi from mice treated with AAV-sFKN, AAV-GFP or control untreated tTA mice after staining by the Gallyas method for neurofibrillary tangles (left) or by immunostaining for pSer396 (right). Neurons expressing tau are visible in the CA subfield as dark reaction product. Scale bar = 200 μm. **b** Percentage area occupied by Gallyas reaction product was quantified on 8 sections per mouse, averaged to yield a single value per mouse, then averaged over all mice per treatment condition. Although there was a genotype effect with rTg4510 mice displaying tangles and tTA mice lacking tangles, there was no effect of treatment with sFKN compared with GFP. **c** Similar quantitation of pSer396 phospho-tau staining as measured by IHC yielded equivalent conclusions. Data represented as mean ± SEM, *n* = 9–12. *** denotes *p* < 0.001 by Fisher’s LSD

**Fig. 7:**
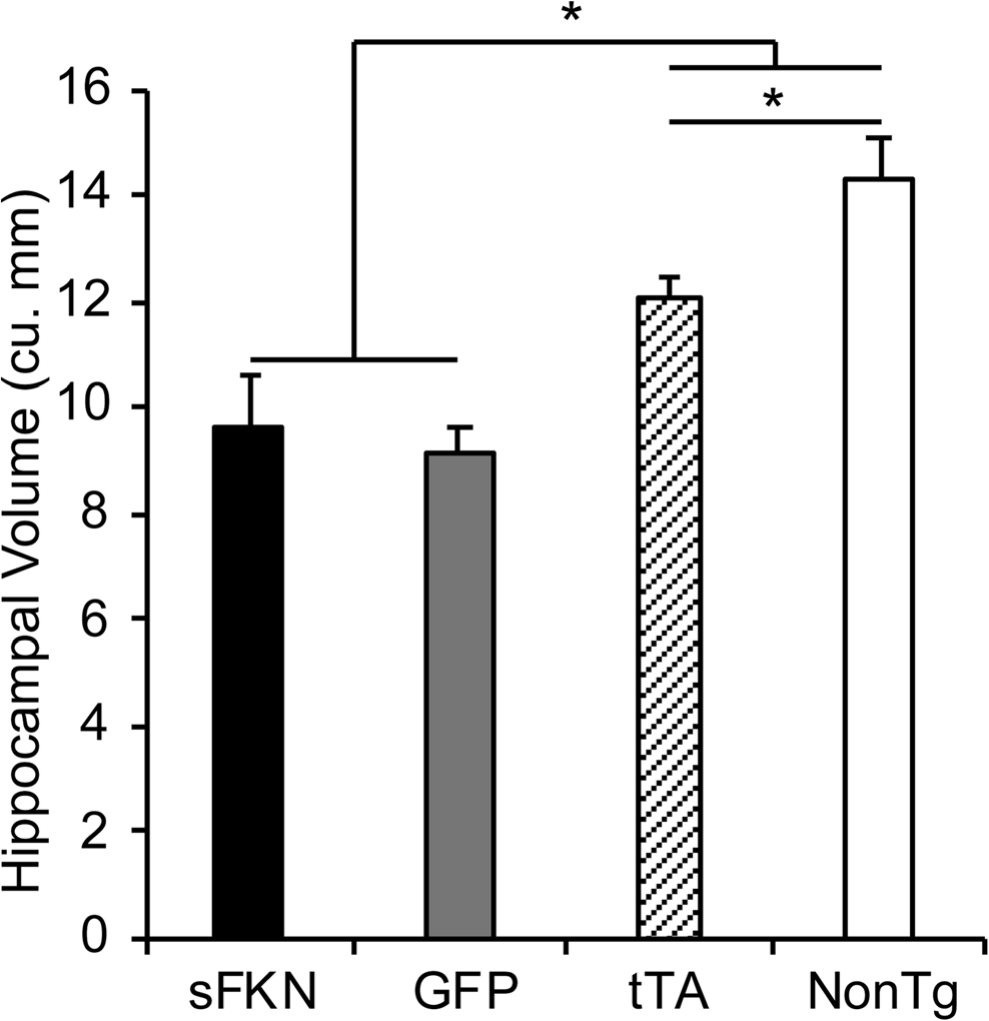
rTg4510s have reduced hippocampal volume. Graph of hippocampal volume. Hippocampal area was quantified across 8 equally spaced sections spanning the hippocampus per mouse, which were averaged to generate one value per animal and finally averaged across all mice in one treatment group. rTg4510s have reduced hippocampal volume compared to uninjected tTA and NonTg controls. Uninjected tTA mice also displayed reduced hippocampal volume compared to uninjected NonTg mice. Data represented as mean ± SEM, *n* = 9–12. * denotes *p* < 0.05 by Fisher’s LSD

We did not observe a significant reduction in either total (H150) or phospho-tau (pSer199/202 & pSer396) in soluble hippocampal homogenates (Fig. 5a, b) in animals treated with sFKN. Unexpectedly, we observed a significant increase in total insoluble tau with a trend for increased forms of phospho-tau (Fig. 5c, d). However, we did not observe significant increases in immunohistochemical staining of pSer396 (Fig. 6a, c) or in Gallyas staining, thought to represent tau tangles (Fig. 6a, b). Furthermore, we did not observe an amelioration of hippocampal atrophy in AAV-sFKN-injected rTg4510s compared to AAV-GFP-injected rTg4510s (Fig. 7). This is not unexpected given the mice would have already shown significant atrophy at time of viral injection.

### Increased Soluble FKN Signaling Did Not Reduce Microglial Markers

**Fig. 8:**
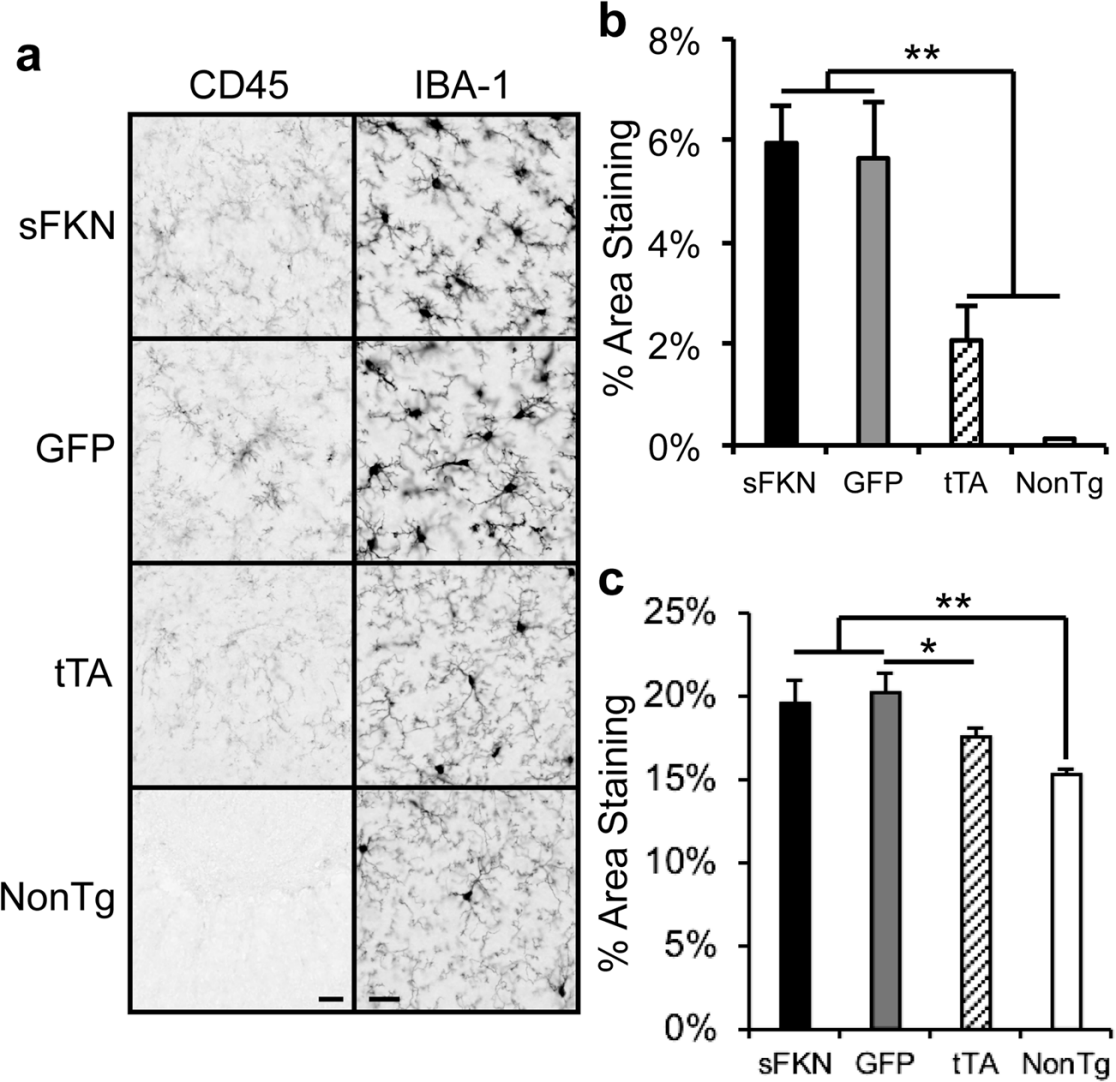
sFKN over expression did not reduce microglial activation. **a** Representative images of CD45 and IBA-1 immunoreactivity. Scale bar = 20 μm. **b** Percentage area staining for CD45. **c** Percentage area staining for IBA-1. Immunoreactivity was quantified across 8 sections per mouse, which were averaged to generate one value per animal and finally averaged across all mice in one treatment group. rTg4510s displayed more area staining of CD45 and IBA-1 than uninjected tTA and NonTg controls, but sFKN did not reduce this staining. Data represented as mean ± SEM, *n* = 9–12. ** denotes *p* < 0.01, * denotes *p* < 0.05 by Fisher’s LSD

**Fig. 9:**
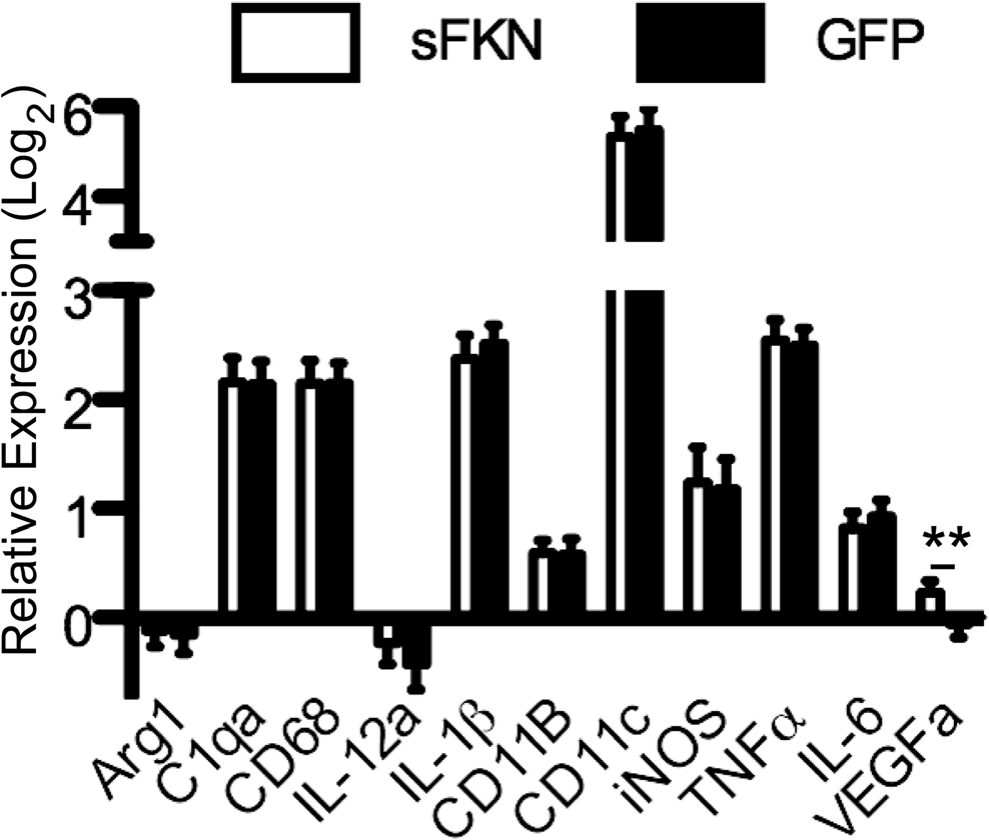
sFKN over expression increased *Vegfa* expression. Graph of log base-2 expression relative to uninjected nontransgenic control mice. Data analyzed using ΔΔC_T_ method. Data represented as mean ± SEM, *n* = 4–7. ** denotes *p* < 0.01 by two-tailed Student’s t test

We have previously shown that CD45, a marker of microglial activation, was elevated in rTg4510s and that parenchymal sFKN over expression reduced this marker (Nash et al. 2013). However, we did not observe a significant reduction in CD45 staining in animals over expressing sFKN in this experiment (Fig. 8a, b). Furthermore, we did not observe a reduction in IBA-1, a pan-marker for microglia in animals over expressing sFKN (Fig. 8c). When we examined expression of a panel of innate immunity-related genes using RNA from the posterior cortex, we did not observe changes in a number of microglial markers but *Vegfa* was significantly elevated in AAV-sFKN-injected rTg4510s compared to control injected rTg4510s (Fig. 9).

## Discussion

Microglia play a key role in Alzheimer’s disease pathogenesis. Previous studies showed that increasing microglial activation exacerbates tau pathology while reducing microglial activation can reduce tau pathology (Cho et al. 2011; Herber et al. 2007; Lee et al. 2010a, 2010b). We previously demonstrated that increasing soluble fractalkine agonism in the rTg4510 mouse model of tauopathy reduced pathology and neurodegeneration. However, the treatment was administered at a preventative timepoint, with administration of virus prior to significant tau pathology formation (injection at 3 months of age). In this study we wanted to examine if sFKN could have beneficial effects when administered at a more therapeutic timepoint, by administering virus after significant disease pathology and memory deficits had already been established (injection at 5 months of age). This would reflect a more clinically relevant setting where diagnosed patients would already exhibit significant tau deposition. The current study also differed from our previous work in that our delivery method in the previous study targeted only the hippocampus with injections directly into the parenchyma; here we sought to maximize treated brain regions while minimizing the number of injections required with the use of AAV4. We envisioned that intracerebroventricular (ICV) injections would reduce tissue damage to brain regions already burdened with pathology, neuron loss, and gliosis while maximizing distribution of the therapeutic gene product, another key consideration for translation to the clinic.

Previous studies showed the rAAV4 vector can efficiently infect cells of the lateral ventricle (Davidson et al. 2000a; Liu et al. 2005). More importantly, it has been reported that ICV and intrathecal delivery of AAV4 and AAV2 vectors encoding lysosomal enzymes, which were also secreted from transduced cells, has proven very effective in providing therapeutic levels of enzyme throughout the CNS in adult mouse models of lysosomal storage disease (Liu et al. 2005; Watson et al. 2006). ICV delivery of an AAV4 vector encoding hIFN-β has also been shown to be an effective approach to curb glioblastoma tumor growth (Meijer et al. 2009). These data suggest that secretion of sFKN from a lateral ventricle injection may be sufficient to provide therapeutic levels of sFKN protein throughout the CNS. In this study, we show that AAV4 sFKN infected the cells lining the lateral ventricles and increased sFKN levels in the CSF and hippocampus of injected mice. We observed sFKN expression in cells throughout the ventricular system including the cerebral aqueduct, 3rd and 4th ventricles. Using this method, we achieved an approximately 2-fold increase of soluble FKN over endogenous levels.

Previous reports have implicated fractalkine signaling in neuropathic pain in the spinal cord (Clark and Malcangio 2012; Milligan et al. 2004). Since we secreted sFKN into the CSF, potentially increasing FKN signaling in the spinal cord, we were concerned this may have detrimental effects and thus examined the animals for thermal hyperalgesia on a hot plate. Animals over expressing sFKN had no change in latency to hind paw withdrawal as compared with GFP-injected controls (Fig. 3), indicating that either the levels of sFKN reaching the spinal cord are insufficient to cause hyperalgesia or the species (ADAM 10/17 product) of soluble FKN (aa 1–336) we over expressed does not participate in mediating neuropathic pain. The latter would be consistent with the data published by Clark and Malcangio (2012). They demonstrated that intrathecal administration of a peptide of the chemokine domain (amino acids 1–110 only) results in mechanical hypersensitivity whereas an injection of the sFKN protein does not alter sensitivity (Clark and Malcangio 2012). Furthermore, induction of allodynia in spinal lesion models has been shown to be mediated, in part, by FKN and requires cathepsin S (Clark et al. 2007, 2009). These findings implicate the cathepsin S cleavage product of FKN, which migrates at a lower apparent molecular weight by SDS-PAGE than the ADAM 10/17 cleavage product used in the current study, in the induction of neuropathic pain (Fonovic et al. 2013).

Examining the behavioral phenotype of rTg4510s, we observed FKN expression above basal levels ameliorates several behavioral abnormalities. As reported previously, rTg4510s are known to be hyperactive and this hyperactivity may be due to pathological tau (Joly-Amado et al. 2016). Here, we observed a significant reduction in locomotor activity as measured in both the open field and Y-maze (Fig. 2). We also observed a significant improvement in novel mouse recognition, however, not novel object recognition (Fig. 2). This may be due to the greater motivation to interact with another mouse as opposed to an inanimate object (Brownlow et al. 2014). In the RAWM task, we observed that increased sFKN levels significantly improved hippocampal-dependent spatial learning and memory (Fig. 4). Animals over expressing sFKN made significantly fewer errors on Day 2 of the task than GFP-injected rTg4510s. However, we observed no improvement in the Reversal task, suggesting that this spatial learning improvement was partial. This may be due to the severe pathology that these animals already exhibited prior to start of sFKN treatment. This would suggest that earlier intervention, prior to significant neuropathology and neurodegeneration, may be a more beneficial therapeutic strategy.

We believe that the improvements we observed in behavior are due to alterations in the inflammatory milieu brought about by sFKN over expression. Previous studies have demonstrated that *Cx3cr1−/−* mice have deficits in cognition and neurogenesis that was reversed with IL-1 Receptor Antagonist (IL-1RA) administration (Bachstetter et al. 2011; Rogers et al. 2011). Microglial-specific cytotoxicity in an adoptive transfer model was also blocked by genetic ablation of IL-1 signaling (Cardona et al. 2006). Similarly, tau hyperphosphorylation induced by adoptive transfer of activated microglia from LPS-challenged hTau;*Cx3cr1−/−* mice into naïve wild-type mice was blocked by administration of the IL-1R antagonist Kineret (Maphis et al. 2015). Antagonism of IL-1R has also been shown to improve cognitive deficits in the 3xTg model of AD (Kitazawa et al. 2011). Finally, a recent report showed that activated microglia can induce a neurotoxic phenotype in astrocytes, that these activated astrocytes are present in AD tissue, and blockage of the transformation of astrocytes into neurotoxic astrocytes can be neuroprotective (Liddelow et al. 2017). Given that FKN agonism reduces pro-inflammatory gene expression, FKN agonism may promote an environment more permissive to learning and memory (Lyons et al. 2009).

Besides cytokine secretion, microglia are also directly involved in synaptic homeostasis. Depletion of microglia caused cognitive deficits and removal of microglial-derived brain derived neurotrophic factor recapitulated these effects, implicating microglial neurotrophic support in learning and memory (Parkhurst et al. 2013). Furthermore, microglia are involved in synaptic engulfment, both in disease and homeostasis (Hong et al. 2016; Schafer et al. 2012). Microglial dysfunction and senescence have been implicated in AD and clearance of senescent glial cells has recently been shown to improve recognition memory in the PS19 model of tauopathy (Bussian et al. 2018; Miller and Streit 2007). Increased FKN agonism may return microglia to a homeostatic state, facilitating learning.

Interestingly, we did not observe changes in microglial pan markers (CD45 and IBA-1), but this is consistent with what we have observed in previous studies in Parkinson’s disease where we showed a neuroprotective effect of sFKN over expression but no concomitant reduction in MHC-II expression (Nash et al. 2015). A likely explanation is that we are altering the microglial state with the addition of sFKN rather than decreasing the level of activation, as these markers can be increased in both proinflammatory and alternative activated states (Lee et al. 2013). We believe that the sFKN subtly altered microglial activation in a such a way that is more permissive to learning without altering expression of either CD45 or IBA-1. We would predict that this altered state would be less pro-inflammatory and possibly more neuroprotective.

To further investigate alterations in microglial activation, we examined a panel of immune-related genes by qPCR and observed a significant increase in *Vegfa* expression in animals over expressing sFKN. While vascular endothelial growth factor (VEGF) is most known for angiogenesis, it can also impact neurogenesis and promote neuron survival. VEGF has been shown to protect primary hippocampal neurons from excitotoxicity in vitro and promote neurogenesis in vivo (Jin et al. 2002; Matsuzaki et al. 2001). In neurodegenerative diseases, VEGF has been shown to be neuroprotective in Parkinson’s disease, amyotrophic lateral sclerosis, and Alzheimer’s disease models (Dodge et al. 2010; Religa et al. 2013; Tian et al. 2007). Neuroprotective effects are mediated both by direct anti-apoptotic signaling in neurons, neurogenesis, and through increased trophic support (Jin et al. 2001, 2002; Storkebaum and Carmeliet 2004; Sun et al. 2003). We hypothesize this increase in trophic support improved neuronal function, which may explain the improvement in cognitive performance observed in this study. Closer examination of the microglial profile with sFKN agonism is the focus of our current investigations.

rTg4510 mice begin to show tau pathology as early as 3–4 months of age with behavioral deficits starting as early as 2.5 months (Dickey et al. 2009; Santacruz et al. 2005). By 6 months of age, rTg4510s have significant insoluble tau deposits and marked neuron loss and behavioral deficits. Furthermore, accumulation of insoluble tau species plateaus from 5.5 months of age onward, as does accumulation of a 64 kDa soluble phospho-tau species (Dickey et al. 2009; Santacruz et al. 2005). Examining the tau pathology in the sFKN treated animals, we observed no significant change in soluble total tau or soluble phospho-tau species, but interestingly we did observe a significant small increase in insoluble total tau in animals over expressing sFKN. When we examined tau pathology by immunohistochemical means, we observed no increase in pSer396 phospho-tau and no increase in Gallyas-positive tau tangles. The inability of sFKN over expression to slow tau pathology here is possibly due to achievement of stable, plateau levels of pathology already present at the time of injection (Dickey et al. 2009; Santacruz et al. 2005; Spires et al. 2006). Alternatively, it may also require higher elevations of sFKN than we obtained here to reduce the accumulation of tau in these mice.

It is also important to note that our results are similar to those reported where the tau transgene expression was suppressed with doxycycline at 5.5 months of age (Ramsden et al. 2005; Santacruz et al. 2005). The mice on doxycycline improved in spatial learning tasks at 7 and 9.5 months compared to those without doxycycline, but the treated mice had no reductions in neurofibrillary tangles, PHF-1 positive neurons, or neuron number in the CA1 subfield (Santacruz et al. 2005). The recovery of memory in this mouse model, by doxycycline and now sFKN treatment, suggests that irreversible structural degeneration may not be responsible for initial memory deficits and that there is potential to recover some cognitive function in early stages of the disease. Indeed, this uncoupling of insoluble tau deposition from neuron loss and cognitive deficits is not unique to this model, as NFT formation does not correlate well with neuron loss in the human brain (Berger et al. 2007; Gomez-Isla et al. 1997; Spires-Jones et al. 2011). This could open the door for some medications to have profound improvements in quality of life for patients and delay the need and reduce the cost of extensive residential care. It has been estimated that a five-year delay in AD could lead to a 40% reduction in costs by 2050 (Zissimopoulos et al. 2014).

We have previously demonstrated that sFKN can be beneficial in a preventative approach in rTg4510 mice with administration at three months of age, prior to significant pathology. Here we shown that delivery of sFKN, using AAV4, can have a beneficial cognitive effect in rTg4510 mice with advanced pathology, at five months of age. In this study, we show the utility of delivering AAV4 to the lateral ventricles and over expressing a soluble immunomodulator into the CSF to achieve broad delivery of a therapeutic gene product to treat tauopathy. We have shown that this delivery method can meaningfully increase sFKN concentrations in the hippocampus as well as ameliorate behavioral deficits in the rTg4510 mouse model of tauopathy with advanced tau pathology. These data suggest that immunomodulation could have significant benefits in AD patients which already show tau pathology.
